# Patient-Reported Outcome Measures for Addiction Treatment: Development of a Digital Health Intervention using a Patient-Centered Approach

**DOI:** 10.1192/j.eurpsy.2025.977

**Published:** 2025-08-26

**Authors:** M. Pellicer-Roca, J. I. Mestre-Pintó, F. Fonseca, G. J. Gil-Berrozpe, M. J. Cuesta, C. Tamarit Francés

**Affiliations:** 1 Research Network in Primary Addiction Care (RIAPAd), -; 2 Addiction Research Group (GRAd), Neuroscience Research Program; 3 Universitat Pompeu Fabra; 4 Hospital del Mar Research Institute; 5 Institut de Neuropsiquiatria i Adiccions (INAD), Parc de Salut Mar; 6 Medicine and Life Sciences (MELIS), Universitat Pompeu Fabra, Barcelona; 7 Department of Psychiatry, Complejo Hospitalario de Navarra; 8 Navarra Institute for Health Research (IdiSNA), Pamplona, Spain

## Abstract

**Introduction:**

Substance Use Disorders (SUDs) are highly prevalent and substantially contribute to the global burden of disease, negatively impacting individuals’ health, well-being, and social functioning. While SUD treatments have demonstrated effectiveness in reducing substance use and related behaviors, there has been a growing emphasis on including subjective outcome measures, recognizing the importance of patients’ perspectives on their well-being. This shift has driven the development of Patient-Reported Outcome Measures (PROMs), which are progressively being integrated in healthcare to enhance patient-centered care, patient-provider communication and shared decision-making.

**Objectives:**

This study aims to develop a PROMs Digital Health Intervention (PROMs-DHI) with the potential to improve the quality and effectiveness of outpatient addiction treatment services.

**Methods:**

PROMs-DHI includes the electronic adaptation of ICHOM’s standardized set for addictions using REDCap software and feedback created by Tableau on individual PROMs scores for patients and clinicians. PROMs-DHI will be assessed through mixed methods following a patient-centered design. Quantitative data will be collected using the System Usability Scale (Brooke, 1986), while qualitative data will be gathered through 5 focus groups with patients and 5 with healthcare professionals from collaborating addiction treatment centers. This strategy will yield valuable insights into the experiences and perspectives of both groups in utilizing the PROMs-DHI, offering a deeper understanding of their interactions and outcomes.

**Results:**

It is expected that patients will perceive a positive effect of the usage of the PROMs-DHI, gaining insights into their progress, reinforcing communication with clinicians, and enhancing self-management. Clinicians are expected to find the PROMs-DHI beneficial for managing patient care, profiting from the ability to monitor patient outcomes in real-time, receive notifications when values exceed critical thresholds, and access detailed reporting tools to inform their treatment decisions. This would enable them to provide more targeted and effective care, ultimately leading to improved patient outcomes.

**Conclusions:**

Implementing the PROMs-DHI in addiction care could facilitate outcome comparisons, guide service improvement, and ultimately increase the effectiveness and quality of interventions, contributing to a stronger and more sustained recovery for patients undergoing treatment. Providing patients with regular feedback on their outcomes may also have a therapeutic effect in itself, promoting self-awareness, motivation and empowerment to manage their recovery. Furthermore, PROMs can support healthcare professionals in making well-informed treatment decisions that are tailored to each patient’s needs, thereby strengthening the impact and sustainability of addiction interventions.

**Disclosure of Interest:**

None Declared

